# Are We Losing the Final Fight against Cancer?

**DOI:** 10.3390/cancers16020421

**Published:** 2024-01-19

**Authors:** Guy Storme

**Affiliations:** Department Radiation Oncology, UZ Brussel, Asfilstraat 20, 9031 Drongen, Belgium; guy.storme@telenet.be

**Keywords:** outcome, relative survival, impact, new drugs, genes

## Abstract

**Simple Summary:**

From a scientific perspective, we are beginning to comprehend the evolution of cancer better, but much remains unknown. The quantity of medications has grown significantly during the past few decades; however, most solid tumor outcomes have not been much affected. Circumstances impact genes that are key players in cancer development. Maybe we should concede that the story of cancer never ends.

**Abstract:**

Despite our increasing understanding of the biology and evolution of the cancer process, it is indisputable that the natural process of cancer creation has become increasingly difficult to cure, as more mutations are found with age. It is significantly more difficult to challenge the curative method when there is heterogeneity within the tumor, as it hampers clinical and genetic categorization. With advances in diagnostic technologies and screening leading to progressive tumor shrinkage, it becomes more difficult over time to evaluate the effects of treatment on overall survival. New treatments are often authorized based on early evidence, such as tumor response; disease-free, progression-free, meta-static-free, and event-free survival; and, less frequently, based on clinical endpoints, such as overall survival or quality of life, when standard guidelines are not available to approve pharmaceuticals. These clearances usually happen quite rapidly. Although approval takes longer, relative survival demonstrates the genuine worth of a novel medication. Pressure is being applied by pharmaceutical companies and patient groups to approve “new” treatments based on one of the above-listed measures, with results that are frequently insignificantly beneficial and frequently have no impact on quality of life.

## 1. New Drugs for Cancer Treatment

Despite the introduction of new technologies and treatments, despite 50 years of developing strategies to fight cancer, and despite a growing understanding of the biology of the disease, we must accept that cancer remains a deadly disease. The natural process of cancer development becomes more difficult to cure. This is because as we age, more mutations are established that lead to new signaling pathways and that circumvent known induced resistance [1]. Cancer is an evolving process that is impacted by mutation and the selection of more suitable clones, which promote development, invade the surrounding tissue, and cause it to break down, eventually leading to metastasis throughout the body [2,3]. The fact that dormant cells, which are invisible when solitary or in clusters, can change over time and interact with extracellular matrix composition, metabolism, and nearby cells to upset the balance and restart the growth process in spite of treatment, leads to late metastases and recurrences that eventually cause patients’ death [4,5,6]. Based on clinical data acquired over the past century, tumor dimension, nodal and/or other organ invasion (TNM) [7], and grading (structure of the tumor inside surrounding tissue: G1,2,3) [8] were developed to classify prognostic groups of patients and treat them according to guidelines. Modern cutting-edge technologies like proteomics and genomics help us better understand how a tumor interacts with the surrounding healthy tissue and how cancer develops. Due to mutations (1) causing resistance, it is difficult to identify heterogeneity within each tumor, which has prevented significant advances in outcomes over the past 20 years [9,10,11]. Between 1980 and 2000, we observed a significant decrease in mortality. Although the pharmaceutical industry optimistically attributes higher survival rates to the development of new, innovative drugs, advances in diagnostic tools, surgical procedures, and radiation therapy have also contributed to trimodal cancer treatment [12,13]. However, the effectiveness and costs of widespread cancer screening are still debated. In breast cancer, Miller et al. [14] found that mammography and echo-radiography were not useful compared to a standard physical examination; they may even be harmful, leading to overdiagnosis and higher costs [15]. The effect of breast cancer screening is apparently insignificant, since calculations from various studies have shown that it takes an average of 10.7 years (4.4–21.6 years of data follow-up) to prevent one breast cancer death out of a thousand women who participated in screening if 1.3 million women are screened in the UK [16]. Overall cancer screening only has life-saving added value after meta-analysis, which shows that only sigmoidoscopy has an effect, but not lung, breast, prostate, or colon cancer screening [17]. Improved survival is ascribed to drugs, but at the same time screening has provided patients with smaller tumors which, on its own, results in longer survival.

Also, during periods of improved drug-induced survival in the Netherlands, the proportion of stage I patients increased from 28% to 41% and the proportion of stage II patients decreased from 51% to 33%, with 91% relative survival [18], which makes it particularly difficult to approve new drugs and evaluate the impact of screening even 83 individual medicines plus the 6 NCI-approved combinations that are available. A recent study of 124 drugs with US Food and Drug Administration (FDA) approval in 374 indications (2003–2020) showed that new anticancer medicines were associated with an improvement of 2.8 months (Inter Quartile Range IQR: 2.0–4.6 months) for overall survival (OS) and 3.30 months (IQR) 1.5–5.6 months) for progression-free survival (PFS) [19].

**Figure 1:**
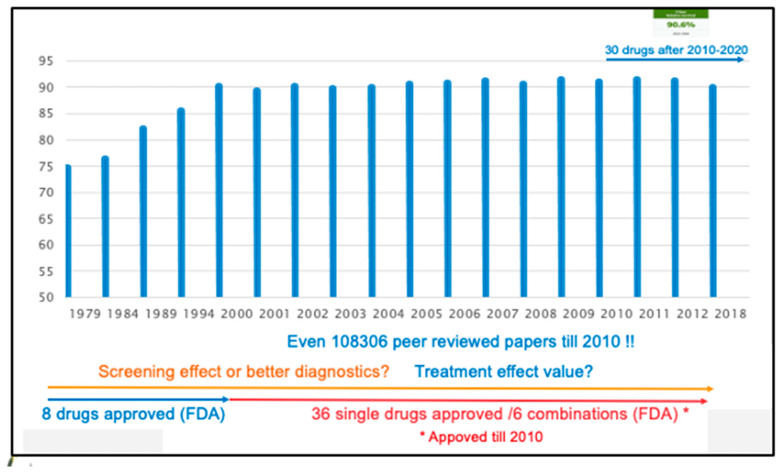
SEER 2020 5-years Relative Survival (RS) % of female breast cancer by year of diagnosis all races [21]. Is added to the SEER data: 8 FDA approved drugs available before 2000 with also screening. 36 approved drugs between 2000 and 2010 which have no effect on RS after 2010. Number of peer reviewed publications till 2010 on all cancer drugs, with screening in same period. Added right upper corner 5-y 90.6% RS SEER (last available data 2012–2018) while in this period 30 new drugs were implemented on the existing ones. Meanwhile 83 single drugs are available (https://seer.cancer.gov/tools/seerrx).

This possibly “marginal” benefit of new cancer drugs is an ongoing debate because the patients in the study are selected and do not correspond to the general population who later receive these drugs inappropriately [20]. From the patient’s point of view, few drugs can be considered “successful”, despite many publications showing mostly significant beneficial differences in breast cancer (see Figure 1).

Observational data show that although “innovative” cancer treatment significantly increases prices for patients and insurance companies it does not always improve overall survival [22]. New breast cancer drugs increase the cost of metastatic disease approximately threefold over 13 years, with no increase in survival [23]. Consider that in RCTs, the costs of new anticancer drugs based on initial indications were as follows: (1) a negative correlation was observed with both disease incidence and prevalence (b = 0.21 and *p* < 0.001); (2) a positive correlation was observed with first-line drugs (26%, *p* = 0.057), gene and cell therapies (176%, *p* < 0.001), hematological cancers (62%, *p* < 0.001), and serious diseases with significant unmet need (6% per disability-adjusted life year, *p* < 0.001); and, finally, (3) a negative correlation with indications was observed in randomized controlled phase 3 trials. The effectiveness of additional indications, clinical evidence, and epidemiology was not positively correlated with prices, for which I refer to paper [22]. As a result, there is a tendency to argue with regulators to consider more readily available endpoints in their reimbursement estimates, ignoring the fact that even significant treatment effects on an intermediate endpoint such as disease-free survival (DFS), or PFS cannot be translated to an OS benefit without proof [24]. However, patients with accelerated FDA clearance were less likely to receive advanced evidence and recommended status in NCCN guidelines compared with conventionally approved cancer drugs. This creates a dilemma. Only 156 (33%) of the 315 oncology indications were rated as a level 1 indication (high level of evidence with uniform panel consensus about randomized trials (>85% of votes by the NCCN)). Compared with regular approval indications, those who received accelerated approval were less likely to be on the list of preferred treatment options (58% vs. 40%; *p* = 0.008) and assigned grade 1 rate (47% vs. 3%; *p* < 0.001) as defined by the World Health Organization Codex for Anatomical Therapeutic Chemicals. Even more questionable is the fact that 8 (38%) of the 21 rapidly approved indications are still within the NCCN guidelines, and most (6/8) have a level 2A evidence rating (lower-level evidence with uniform panel consensus of clinical retrospective trials >85% of votes by the NCCN). Even more questionable is that most of the criteria for routinely approved drugs were based on weaker evidence [25]. Furthermore, one in five (n = 19/93) cancer drug indications approved under the FDA’s expedited approval process showed improved overall patient survival in confirmatory studies. Requirements for confirmatory studies may need to be re-evaluated to obtain more clinically meaningful data [26]. Most trial-level validation studies in oncology find moderate correlations with survival when associated with surrogate outcomes. All validation studies use only a portion of the available research. There is scant evidence supporting surrogate endpoints in oncology, as more than half of the evidence is underpowered [27]. The FDA has approved a higher proportion of drugs since 2008 than ever before, and cancer drugs are approved based on surrogate endpoints that have poor correlation with OS. Therefore, we may approve many expensive and toxic drugs with no evidence that they improve OS. For patients, post-marketing studies are essential, and it is paramount that patient actions are enforced by regulatory authorities [28]. Regulators may follow Europe’s example, as 4444 medicines approved under conditional marketing authorization must renew their approval annually and meet specific requirements to receive full approval [29]. You may also have questions about how drugs are approved and how they affect your operating system and quality of life. A retrospective analysis of 48 anticancer drugs for 68 indications approved by the European Medicines Agency between 2009 and 2013 found that only 35% of the cancer drugs showed a significant increase in survival, with only one showing a benefit after 8 months (median 27 months). The improvement in quality of life was only 10%. Of the indications for which there was no evidence of survival at market launch, only 7% showed an increase in lifespan and 11% showed an improvement in quality of life. After a minimum follow-up of 5.4 years, only 51% showed significant improvement in OS or quality of life. Of the 23 indications related to survival benefit that could be assessed using the ESMO-MCBS tool, less than half (11/23, 48%) of the benefits were judged to be clinically important [30]. The impact of pharmaceutical company involvement in most clinical trials is publicly debated and clearly impacts the oncology community. The commitment to transparency in legal proceedings is questionable. Two-thirds received at least industry funding, and more than half received industry funding only. Approximately 60% had authors from the industry, and 20% were analyzed solely by the industry [31]. Few data were easily accessible to others, and industry-funded non-randomized trials were favorable to sponsors, whereas exclusive industry-funded trials and industry-affiliated authors were positive for sponsors [32]. The Accelerated Approval (AA) program has successfully accelerated regulatory approval of new cancer drugs based on surrogate endpoint data. Because AA-approved drugs often take a long time to review, it is unclear whether the AA program will facilitate overall drug development, including clinical efficacy testing. Early identification of indications for AA will reduce the time it takes for our patients to receive unproven medicines and will give patients in the EU and Japan faster access to innovative new medicines. It is recommended to initiate confirmatory testing [33]. This means that most new cancer drugs, with little overall survival benefit and questionable impact on patient quality of life, are associated with high costs to society and lasting economic impact on patients [33]. Because we use surrogate endpoints such as disease-free survival (DFS), progression-free survival (PFS), metastasis-free survival (MFS), event-free survival (EFS), and overall survival (OS), we advocate establishing standards for quality of life (QoL) and treatment benefit threshold criteria. It is surprising that in studies of systemic therapy for gastrointestinal cancers, the primary endpoint was DFS, PFS, or EFS in 62% of cases and OS in only 32%. This is in contrast to radiotherapy trials where, with rare exceptions, overall survival (OS) was the primary endpoint [34]. In my opinion, OS is essential as an endpoint and, if significant benefit is observed, should be extended to population relative survival (RS) before reimbursement is allowed. What is our argument? If the average human lifespan is human 80 years (=960 months), then the average overall survival benefit of 2–8 months corresponds to only a 0.229% increase in a lifespan. The proposal for the acceptance of new treatments should give at least an overall survival benefit of 2 years, including good quality of life, which corresponds to extending a life by 2–5%. If starting a new trial without using the same molecular approach, oncologists should not inform patients about such calculations. Five-year relative survival rates for breast cancer from SEER data from 2000 to 2018 show values of 90.1% in 2000, 90.6% in 2018, and 89.4% or 91.2% in 2008 (see Figure 1). It is difficult to draw conclusions about the increase in benefit of RS given the small differences between the lowest and highest values are not linear over time [35]. The majority of research, as well as institutions like the FDA and EMA, are deficient in adopting specific retention criteria [36].

We might draw the conclusion that, despite amazing research efforts over the past 20 years to better understand cancer pathways, therapeutic interactions, and screening methods, very little progress has been made in improving population outcomes.

## 2. Therapeutic Consequences of Gene Signatures

Impressive experimental and clinical results regarding promising genetic features such as in breast cancer have not translated into benefits to patient outcomes. Although no therapeutic drugs have been developed for acquired somatic abnormalities, genetic analysis has enabled new molecular stratification of the breast cancer population by analyzing the genome and transcriptome structure of 2000 breast cancers [37]. Targeting the BRCA1 gene, which was discovered more than 30 years ago, has not yielded an overall survival benefit in breast cancer, yet researchers are looking for gene combinations to find a solution. In our opinion, it is unlikely that a therapy targeting a combination of genes will be found. Available data suggest that a 70-gene signature may help determine whether breast cancer patients require adjunctive systemic treatment. This ability has also been demonstrated using good and bad clinical data related to genetic characteristics, including WHO 0–1, adequate bone marrow reserve, renal function, liver function, and specific heart disease or medical conditions, which were applied in [38]. It is known that as we age, genes involved in cancer development continue to evolve [1]. A long-term genome-wide association study in European, African, Asian, and Hispanic men found a 57% increase in the number of non-European cases compared to previous genome-wide association studies in prostate cancer. It is shown that 187 new prostate cancer risk variants were identified, bringing the total number of risk variants to 451. Externally replicated multi-ancestral genetic risk scores (GRSs) are associated with risks ranging from 18 for men of African descent to 22 for men of European descent, with African men having higher risk of invasive and non-invasive disease. The risk was high (*p* = 0.003) [39]. Increasing evidence suggests that the microbiota influences cancer susceptibility in part through its vast metabolic capacity and profound impact on immune cell function, and multifactorial factors influence genetic evolution. Although 15–20% of cancer cases can be caused by the microbiota, related studies cannot distinguish whether this microbiota alteration is a cause or a consequence of cancer [40]. The microbiota can alter cancer susceptibility and progression through various mechanisms, including the regulation of inflammation, the induction of DNA damage, and the production of metabolites involved in carcinogenesis or tumor suppression, thus impacting therapy [41]. Manipulating the microbiota can influence the outcome of cancer treatment [42]. Intestinal epithelial cells, intestinal mesenchymal cells, immune cells, and gut microbiota are known to maintain colon homeostasis. Various studies have shown that bacteria promote the progression of CRC through the recruitment of macrophages and the activation of helper T cells. Various microorganisms, such as pathogenic bacteria, probiotics, and fungi, exert or reduce tumorigenic factors within the host through interactions [43]. Genetic alterations in colorectal cancer associated with colitis are different from genetic alterations in sporadic colorectal cancer [44] and are due to different microbiota interactions.

To quote Watson (1962 Nobel Prize), “DNA has revealed the cause, but it may never reveal the cure”.

## 3. Is There Some Light at the End of the Tunnel: Is Immunotherapy a Glorious Future?

Looking at innovative therapeutic approaches in recent years, immunotherapy seems to have risen to the forefront in the early 2000s [45]. The first one is Bacillus Calmette–Guérin (BCG), which is used for non-muscle invasive bladder cancer [46]. Numerous immunotherapies of different classes become available using checkpoint inhibitors, cytokines, chimeric antigen receptor T cells and T cell receptor T cells, costimulatory receptor antagonists, and dendritic cell-based immunostimulatory cytokines. Immunomodulatory monoclonal antibodies, oncolytic viruses, and pattern recognition receptor agonists were already described by Galluzzi [47]. After some useful evidence was obtained in preclinical studies, the number of clinical trials increased. However, its widespread use is hampered by a number of side effects, including autoimmunity; non-specific inflammatory diseases such as immune thrombocytopenia, myocarditis, and Guillain–Barre syndrome; and rare events such as thrombocytopenia, psoriasis, IgA nephropathy, and systemic lupus erythematosus as reported for COVID-19 vaccination [48]. However, as these treatments have serious side effects as mentioned above, controlling the regulation of the immune system remains a key challenge in the widespread implementation of immunotherapy for cancer. The latter events have rare occurrences and do not prevent this vaccine from being used as a vaccine against human papillomavirus, developed by Zur Hausen and reducing the incidence of cervical cancer [48]. The safety of 9vHPV vaccination [49] led to a significant reduction in the incidence of new cervical cancers, as observed between 2000 (8.8/100,000) and 2020 (6.18/100,000) PORT [50]. Considering the positive effects of these immunotherapy drugs, the question arises whether cancer can be cured in combination with available chemotherapy, radiotherapy, and surgery. At least since the approval of immune checkpoint inhibitors in 2011, it has been observed that melanoma survival rates have increased. Accordingly, the SEER database shows a 4% increase in RS and a 20% real survival after the approval of immunotherapy for metastatic disease with immune checkpoint inhibitors (ICIs) [51,52]. It should also be considered that screening programs influence survival rates by detecting thinner melanomas [53], contributing in part to this 20% reduction in mortality over 15 years [54]. With all the different drugs available, a new problem arises: Medication Reconciliation (MR) plays a key role in identifying 51% of the banned or monitored drugs that were initially missed by oncologists but were identified by clinical pharmacists [55].

While there are some encouraging signs in this section, further research and data are still required to determine the overall impact on a cure.

## 4. Palliation

The majority of cancer-related deaths are caused by metastases. However, metastasis is understood to be a gradual, evolving process. These various systemic diseases are beginning to be more effectively treated. The success of these events depends on unrestricted proliferation, clonal selection, the ability of metastatic cells to dynamically transition to different states, and the ability to co-opt immune environments and tissues [56]. Once metastases are discovered, all patients require a palliative approach with or without further treatment, which often ends up doing more harm than good to the patient. There is evidence that the involvement of palliative care teams improves the care provided by oncology teams. To date, five clinical models of palliative care are available and are positively impacting patients, including outpatient clinics, patient consultation groups, acute palliative care units, community palliative care, and hospice care [57]. The median survival was longer for patients who received early palliative care, even though fewer patients received aggressive end-of-life (11.6 months vs. 8.9 months, *p* = 0.002) care (33% vs. 54%, *p* = 0.05) [58]. Money spent without a survival benefit for cancer patients could be used to optimize palliative care and infrastructure, as well as essential health workers. In the words of Soto Perez de Celis: “What if the benefits of early supportive care were instead delivered by an innovative (and likely expensive) drug-antibody conjugate, perhaps called paliatuzumab-supportercan, would it also take decades for it to become the standard of care?” [59]. Although we now have a better understanding of the complexity of cancer, no significant progress has been made in this field. Most studies have obvious biases in their selection criteria, which focus on lower-risk study populations rather than the larger population covered by SEER data. In addition to the underuse of control groups (crossover, post, etc.) in clinical trial design, there is also the problem of progressive treatment and inappropriate comparison products. As a result, our health care authorities need to face reality and the fact that not all RCTs are representative of our clinical reality due to the selected patients not being representative of the general population and the control arms not being representative of the newest treatment guidelines.

More early palliative approaches in metastatic patients should be investigated for the benefit of our patients and society in order to increase quality of life, survival, and treatment costs overall.

## 5. Conclusions

One might conclude that despite recent advances in understanding the mechanisms behind cancer progression, cancer remains one of the leading causes of death worldwide. Cancer treatment is a complex and multistep process. In addition to discovering and developing new medicines, we must focus on providing patients with the best possible treatment at a price that is acceptable to them and society as a whole. Clinical trials must follow strict, well-defined guidelines to demonstrate long-term improvements in overall survival and quality of life, creating a better future for all patients and society.

## Data Availability

The data presented in this study are available in this article.
